# Effects of Parameter Variations Generated by Pumping on LNAPL Migration in the Aquitard: An Analytical and Experimental Study

**DOI:** 10.3390/toxics13060471

**Published:** 2025-06-02

**Authors:** Yue Su, Yong Huang, Huan Shen, Xiaosong Dong, Xiaochang Sun, Zhimin Fu

**Affiliations:** 1 School of Earth Sciences and Engineering, Hohai University, Nanjing 210098, China; suyue@hhu.edu.cn (Y.S.);; 2 Department of Earth and Environmental Sciences, University of Waterloo, Waterloo, ON N2L 3G1, Canada; 3 Faculty of Management Engineering, Huaiyin Institute of Technology, Huaian 223003, China; shenh@hyit.edu.cn; 4 College of Hydrology and Water Resources, Hohai University, Nanjing 210098, China

**Keywords:** LNAPL migration, aquitard, analytical solutions, parameter variations, laboratory experiments

## Abstract

The parameter variations in the aquitard have an important influence on the migration laws of contaminants in the aquitard. In order to study the influence of dynamic changes in parameters during pumping on the migration laws of Light Non-aqueous Phase Liquid (LNAPL) in the aquitard, the one-dimensional consolidation and groundwater flow equations for the aquitard were employed to derive the governing equations for the migration of LNAPL in the aquitard. A self-designed experimental platform was developed to investigate the effects of the pore water pressure, consolidation deformation, and pumping rate on LNAPL migration during pumping. The laboratory experimental results indicated that during pumping, the migration behavior of LNAPL in the aquitard typically exhibited a trend toward the pumping well and the overlying aquifer. The closer to the pumping well, the greater the change in the pore water pressure, the greater the amount of consolidation deformation, the earlier the state of densification, and the slower the migration rate of LNAPL in the aquitard. The nearer to the bottom of the aquitard, the larger the amount of consolidation deformation in the aquitard and the slower the migration rate of LNAPL in the aquitard. Also, the pumping rate had an important influence on groundwater flow movement and contaminant migration. The characteristics of parameter variations in the aquitard and laws of LNAPL migration during pumping were systematically studied and analyzed; these research results can provide a reference for the prediction and remediation of LNAPL in contaminated sites.

## 1. Introduction

Groundwater is an important and indispensable component of water resource systems. As far as the contamination of aquifers is concerned, contamination of the aquitard is characterized by being more hidden and difficult to reverse [1,2,3]. Once the aquitard becomes contaminated, it is challenging to treat and restore due to its inherently slow renewal cycle [4,5,6]. Among the various types of groundwater contaminants, Light Non-aqueous Phase Liquids (LNAPLs) have low degradability and are difficult to eliminate. Once they enter the soil and groundwater environment, they persist for extended periods, posing significant threats to human health and ecosystems [7,8,9,10].

In recent decades, several researchers have employed numerical analysis, indoor experiments, and numerical simulation methods to investigate the migration laws of LNAPLs in the aquitard [11,12,13]. The sudden decrease in the vertical migration rate of LNAPLs in stratified soil media at the fine–coarse interface is partly due to differences in capillary pressure between soil layers, as found by Pan et al. [14]. Oostrom et al. [15] analyzed the distribution of pollutants from a leakage source to soil and groundwater by simulating their transport in an indoor two-dimensional sandbox with seepage from a fluctuating water table. The experiment demonstrated that pollutants initially stored in the air pocket moved vertically with the water under the influence of seepage from the fluctuating water table. During the water table rise, some pollutants in the upper contaminated area dissolved in the water, leading to a significant increase in the contaminants’ concentration in the groundwater. Additionally, the fluctuation of the water table resulted in changes in the concentration of gases in the soil pore space. Chapman et al. [16] used fluorescent dye tracers in tank experiments to visualize mass distribution within multiple low-permeability lenses and compared the dispersion rates among numerical models. Yu et al. [17] conducted a sensitivity analysis on factors affecting LNAPL migration, including the attenuation rate, groundwater depth, hydraulic gradient, and porosity. Zuo et al. [18] conducted a two-dimensional experiment to study the flow and distribution characteristics of LNAPL in the vadose zone under two different stratified geological conditions. The results showed that the vertical infiltration rate of the LNAPL front in medium-coarse sand was approximately an order of magnitude higher than that in low-permeability, fine sand. Additionally, LNAPL accumulation occurred at the fine–coarse interface due to the effect of the capillary barrier. In conclusion, in stratified soil media, the capillary barrier formed at the fine–coarse interface impedes the vertical migration of LNAPLs. Currently, in the study of LNAPLs in aquifers, most researchers focus primarily on the properties of the LNAPL. The investigation of LNAPL migration has been largely centered on fractured and porous media, with comparatively less attention given to the low-permeability characteristics of the aquitard [19,20]. The effect of parameter variations on LNAPL migration in the aquitard is often overlooked. In fact, parameter variations play an important role in LNAPL migration, and the study of parameter variations can enrich the theory of LNAPL multiphase flow migration. It provides theoretical guidance for pollution monitoring of chemically contaminated sites.

Most pumping tests are typically conducted to obtain the hydraulic parameters of the aquifer. The dynamic variations in the aquifer parameters induced by groundwater extraction in contaminated aquifer systems and their impact on contaminant migration have become a focus of research [21,22,23]. Both analytical solutions and experimental simulations have been extensively employed by many experts and scholars to study the process of pumping experiments [24,25]. However, in recent years, pumping tests in contaminated groundwater have also been commonly used as a method for groundwater remediation [26,27,28]. Pumping water will cause significant changes in various parameters in the aquitard, such as the irregular consolidation deformation of the aquitard [29,30], the complexity of the pore water pressure distribution [31], and the difference in the hydraulic permeability coefficient [32]. Most studies tend to simplify the aquitard and do not consider the impact of these changes on LNAPL migration.

Pumping in the aquitard can greatly change the related parameters such as porosity, the pore water pressure, and consolidation deformation. The main objective of this study is to investigate the effects of the pore water pressure, consolidation deformation, and pumping rate on the migration laws of LNAPLs in the aquitard through equation derivations and laboratory experiments. First, the equation for LNAPL migration in the aquitard was deduced by coupling the one-dimensional consolidation deformation equation and the groundwater flow equation. Then, a self-designed experimental platform was developed; the effects of the pore water pressure, consolidation deformation, and pumping rate on the migration characteristics of LNAPLs in the aquitard were analyzed and discussed. Finally, for LNAPL-contaminated sites with pump-and-treat (P&T) technology, a method for evaluating the response of LNAPL migration in the aquitard to parameter variations was determined, which can provide a reference for the treatment and remediation of petroleum-contaminated sites.

## 2. Materials and Methods

### 2.1. Derivation of the NAPL Contaminant Migration Equation

#### 2.1.1. Concept Model and Governing Equation

**Figure 1:**
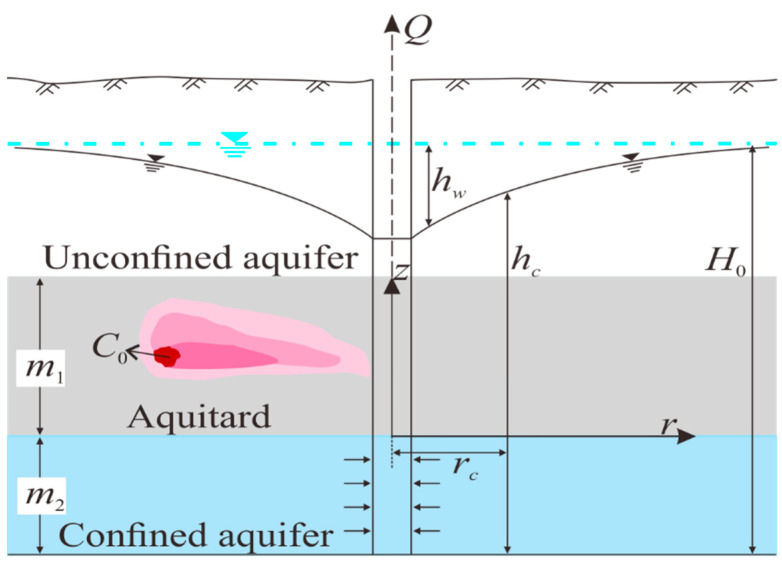
Schematic diagram of the aquifer-aquitard system (1 arrows indicating groundwater flow directions, 2 red areas representing contaminant plume extent, 3 blue areas representing the water extraction layers, 4 blue dashed line indicating the initial groundwater level).

This paper describes the study of the migration characteristics of Non-aqueous Phase Liquids (NAPLs) in the aquitard. Figure 1 depicts the migration of NAPL contaminants in the aquitard. It is a three-layer aquifer system including unconfined aquifer, aquitard, and confined aquifer. A fully penetrated well in this aquifer system has been continuously pumping water at a constant flow rate. NAPLs are dosed into the aquitard. In order to carry out a systematic analysis, the following assumptions are introduced: (1) both the aquifer and aquitard are infinitely extensive and with a uniform thickness, (2) the aquitard layer is homogeneous, (3) the flow in the confined aquifer is horizontal, while the flow in the aquitard is vertical, (4) the total stress at any position of the aquitard remains unchanged during the pumping process, (5) the groundwater level within the underlying aquifer suddenly drops at the initial time and remains unchanged, while the groundwater level within the overlying aquifer remains unchanged, (6) the compressibility of the aquitard changes during deformation of the soil skeleton consolidation induced by pumping, (7) only vertical deformation and vertical seepage take place in the aquitard.

The *z*-axis is defined to be vertical and positive upward, and the origin of the coordinate system is on the axis of the well at the base of the aquitard.

Based on these assumptions, the governing equations for the migration of NAPLs in the aquitard can be obtained [33]:

(1) $$R\frac{\partial C}{\partial t}=\frac{D_{z}}{\theta }\frac{\partial^{2}C}{\partial z^{2}}+\frac{D_{r}}{\theta }\frac{\partial^{2}C}{\partial r^{2}}-\frac{v}{\theta }\frac{\partial C}{\partial r}$$

The initial condition for the aquitard is

(2) $$C\left(r,z,t=0\right)=0$$

The infinite boundary conditions of solute transport in the aquitard are

(3) $$C\left(r\to \infty ,z,t\right)=0$$

(4) $$C\left(r,z,t\to \infty \right)=0$$

The boundary condition of solute transport in the aquitard is

(5) $$C\left(r\to r_{w},z,t\right)=C_{0}$$

(6) $${\left.4\pi r_{w}{Β}_{1}\theta \left(vC-\frac{Dr}{\partial r}\right)\right|}_{r=r_{w}}=QC_{0}$$

(7) $$D_{r}=a_{r}\left|v\right|+D_{0a}$$

(8) $$D_{z}=a_{z}\left|v\right|+D_{0a}$$

where *C* is concentration of Non-aqueous Phase Liquid [ML^−3^]; *C*_0_ is initial concentration of Non-aqueous Phase Liquid [ML^−3^]; *r* is radius distance from the center of the well [L]; *r_w_* is radius of the well [L]; *z* represents vertical distance [L]; *v* is groundwater flow rate of the aquitard [LT^−1^]; *D_z_* and *D_r_* are radial and vertical dispersion coefficients in the aquitard, respectively [L^2^T^−1^]; *D*_0*a*_ = τ*D*_0_, and *D*_0_ is the effective diffusion coefficients in the aquitard; *Da* is the free-water molecular diffusion coefficient of the solute; τ is the tortuosity value (between 0 and 1) in the aquitard; *a_r_* and *a_z_* are the vertical dispersivity values in the aquitard, respectively [34]; $R=1+\frac{\rho_{b}+k_{d}}{\theta }$ is known as the retardation factor of the aquitard; *k_d_* is the equilibrium distribution coefficient [L^3^M^−1^]; *ρ_b_* is the bulk density of the aquitard material [ML^−3^]; *θ* is porosity in the aquitard; and *Q* is the pumping rate [L^3^T^−1^].

Applying the Laplace transform to above equation groups Equations (1)–(8), one has the following for the aquitard [35]:

(9) $$Rs\bar{C}\left(r,z,s\right)=\frac{D_{z}}{\theta }\frac{\partial^{2}\bar{C}}{\partial z^{2}}+\frac{D_{r}}{\theta }\frac{\partial^{2}\bar{C}}{\partial r^{2}}-\frac{v}{\theta }\frac{\partial \bar{C}}{\partial r}$$

(10) $$\bar{C}\left(r,z,s\right)=0$$

(11) $$\bar{C}\left(r\to \infty ,z,s\right)=0$$

(12) $$\bar{C}\left(r,z,t\to \infty \right)=0$$

(13) $$\bar{C}\left(r\to r_{w},z,s\right)=C_{0}$$

(14) $${\left.4\pi r_{w}{Β}_{1}\theta \left(v\bar{C}-\frac{Dr}{\partial r}\right)\right|}_{r=r_{w}}=QC_{0}$$

where *s* is the Laplace transform parameter in respect to *t*, and the over bar implies the variables in the Laplace domain hereinafter.

The finite Fourier transform along the *z*-axis is employed to separate the *r* and *z* components for the solution. The concentration in Laplace domain can be written as follows:

(15) $$\bar{C}\left(r,z,s\right)=\sum_{m=1}^{M}\sum_{n=1}^{N}A_{mn}\left(s\right)\phi \left(r\right)\psi \left(z\right)$$

where A*_mn_*(*s*) are the coefficients in the Laplace domain and *Φ*(*r*) and *ψ*(*z*) are the spatial basis functions of the Fourier transform, respectively, which are used to represent the components along the radial direction *r* and perpendicular to *z*.

Substituting Equation (3) into (9), we obtain

(16) $$Rs\bar{C}\left(r,z,s\right)=\frac{D_{z}}{\theta }\frac{\partial^{2}\bar{C}}{\partial z^{2}}+\frac{D_{r}}{\theta }\frac{\partial^{2}\bar{C}}{\partial r^{2}}$$

Equation (16) is transformed into an ordinary differential equation, and the method of separation of variables is used. Assuming that the solution is in the form of $\bar{C}\left(r,\;z,s\right)=R\left(r\right)Z\left(z\right)$, substituting it into the original equation yields the following:

(17) $$RsR\left(r\right)Z\left(z\right)=\frac{D_{z}}{\theta }Z^{\prime \prime }\left(z\right)R\left(r\right)+\frac{D_{r}}{\theta }R^{\prime \prime }\left(r\right)Z\left(z\right)$$

By dividing both sides by *R*(*r*)*Z*(*z*), we obtain:

(18) $$Rs=\frac{D_{z}}{\theta }\frac{Z^{\prime \prime }\left(z\right)}{Z\left(z\right)}+\frac{D_{r}}{\theta }\frac{R^{\prime \prime }\left(r\right)}{R\left(r\right)}$$

(19) $$\frac{D_{z}}{\theta }\frac{Z^{\prime \prime }\left(z\right)}{Z\left(z\right)}=-\lambda^{2}$$

(20) $$\frac{D_{r}}{\theta }\frac{R^{\prime \prime }\left(r\right)}{R\left(r\right)}=Rs+\lambda^{2}$$

Solving the first equation yields the general solution for *Z*(*z*):

(21) $$Z\left(z\right)=A_{1}\exp\sqrt{\left(\lambda^{2}\theta /D_{z}\right)}z+A_{2}\exp\left(-\sqrt{\left(\lambda^{2}\theta /D_{z}\right)}z\right)$$

Solving the first equation yields the general solution for *R*(*r*):

(22) $$\xi =\sqrt{\frac{\left(Rs+\lambda^{2}\right)\theta }{D_{r}}r}$$

After replacing *r* with *ξ*, we can get

(23) $$\frac{d^{2}R}{d\xi^{2}}+\frac{1}{\xi }\frac{dR}{d\xi }-R=0$$

The above is the Bessel equation, and its general solution is

(24) $$R\left(r\right)=B_{1}J_{0}\sqrt{\frac{\left(Rs+\lambda^{2}\right)\theta }{D_{r}}}r+B_{2}Y_{0}\sqrt{\frac{\left(Rs+\lambda^{2}\right)\theta }{D_{r}}}r$$

Therefore, the general solution of Equation (16) is

(25) $$\bar{C}\left(r,z,s\right)=\left[A_{1}e^{\sqrt{\frac{\lambda^{2}\theta }{D_{z}}}z}+A_{2}e^{\sqrt{\frac{\lambda^{2}\theta }{D_{z}}}z}+B_{1}J_{0}\left(\sqrt{\frac{\left(R+\lambda^{2}\right)\theta }{D_{r}}}r\right)+B_{2}Y_{0}\left(\sqrt{\frac{\left(R+\lambda^{2}\right)\theta }{D_{r}}}r\right)\right]$$

where A_1_, A_2_, B_1_, B_2_, and *λ* are constants.

The derived Equation (25) can describe the changes in concentration of the NAPL migration. The concentration is related to the porosity of the aquitard and the groundwater flow rate. The porosity of the aquitard is inversely proportional to the NAPL concentration. The smaller the porosity, the larger the NAPL concentration. The groundwater flow rate is directly proportional to the NAPL concentration.

#### 2.1.2. Derivation of the Porosity Variation

As the bottom of the aquitard is subjected to groundwater fluctuations caused by pumping, the pore water pressure of the aquitard changes, and the soil skeleton deforms consequently [34,36]. Assuming that the *z*-axis is positive downward, the continuity equation for one-dimensional consolidation in water flow is expressed as follows:

(26) $$C_{v}\frac{\partial^{2}u}{\partial z^{2}}=\frac{\partial u}{\partial t}$$

The initial and boundary conditions for the aquitard are

(27) $$u\left(0,t\right)=0\begin{array}{cc} & \left(0<t<\infty \right) \end{array}$$

(28) $$u\left(H,t\right)=0\begin{array}{cc} & \left(0<t<\infty \right) \end{array}$$

(29) $$u\left(z,0\right)=-\frac{\Delta H\gamma_{w}}{H}z+\Delta H\gamma_{w}\begin{array}{cc} & \left(0<z<H\right) \end{array}$$

Use the separation of variables method to solve Equation (26), assuming that *u* (*z*, *t*) can be expressed as $u\left(z,t\right)=Z\left(z\right)T\left(t\right)$.

The expression for *u* (*z*, *t*) is derived as follows:

(30) $$u\left(z,t\right)=\frac{2\Delta H\gamma_{w}}{\pi }\sum_{n=1}^{\infty }\frac{1}{n}e^{-\left(\frac{n^{2}\pi^{2}}{H^{2}}\right)C_{v}t}\sin\left(\frac{n\pi z}{H}\right)\left(n=1,2,3\cdot \cdot \cdot \right)$$

where $s_{c}=\int_{0}^{H}au\left(z,t\right)dz$. Substituting the expression for *u* (*z*, *t*), one gets

(31) $$s_{c}\left(t\right)=\int_{0}^{H}a\left(\frac{2\Delta H\gamma_{w}}{\pi }\sum_{n=1}^{\infty }\frac{1}{n}e^{-\left(\frac{n^{2}\pi^{2}}{H^{2}}\right)C_{v}t}\sin\left(\frac{n\pi z}{H}\right)\right)dz=\frac{2\Delta H\gamma_{w}}{\pi }\sum_{n=1}^{\infty }\frac{1}{n}e^{-\left(\frac{n^{2}\pi^{2}}{H^{2}}\right)C_{v}t}\int_{0}^{H}\sin\left(\frac{n\pi z}{H}\right)dz$$

The Fourier series expansion is utilized to obtain

(32) $$\int_{0}^{H}\sin\left(\frac{n\pi z}{H}\right)dz=\frac{H}{n\pi }$$

Therefore, the consolidation deformation of the aquitard expression is as follows:

(33) $$s_{c}\left(t\right)=\frac{2\Delta H^{2}\gamma_{w}a}{\pi }\sum_{n=1}^{\infty }\frac{1}{n^{2}}e^{-\left(\frac{n^{2}\pi^{2}}{H^{2}}\right)C_{v}t}$$

where $C_{v}=\frac{k\left(1+e_{0}\right)}{\gamma_{w}a}$.

The porosity in the aquitard can be obtained:

(34) $$\theta \left(t\right)=1-\frac{2}{\pi }\sum_{n=1}^{\infty }\frac{1}{n}e^{-\left(\frac{n^{2}\pi^{2}}{H^{2}}\right)C_{v}t}\sin\left(\frac{n\pi z}{H}\right)$$

where *C_v_* is the consolidation coefficient [L^2^T^−1^], *u* is the pore water pressure in the aquitard [ML^−1^T^−2^], *z* is the vertical distance from the top surface of the aquitard [L], *t* is time [*t*], *γ_w_* is the weight of water [ML^−2^T^−2^], *e*_0_ is the initial void ratio of the aquitard, Δ*H* is the head change of the aquifer, *k*_0_ is the initial hydraulic conductivity of the aquitard [LT^−1^], *k* is the hydraulic conductivity of the aquitard [LT^−1^], *θ* is the porosity of the aquitard, *a* is the compression coefficient of the aquitard [M^−1^LT^2^], *s_c_* is the consolidation deformation [L].

#### 2.1.3. Derivation of the Groundwater Flow Rate

The governing equation for water flow in a confined aquifer and the aquitard can be expressed as follows [37,38]:

(35) $$\frac{\partial^{2}h_{1}\left(r,t\right)}{\partial r^{2}}+\frac{1}{r}\frac{\partial h_{1}\left(r,t\right)}{\partial r}-\frac{\left[h_{0}-h_{1}\left(r,t\right)\right]k}{T^{\prime }}=\frac{S^{\prime }}{T^{\prime }}\frac{\partial h_{1}\left(r,t\right)}{\partial t}$$

(36) $$T\frac{\partial^{2}h_{2}\left(r,t\right)}{\partial z^{2}}=S\frac{\partial h_{2}\left(r,t\right)}{\partial t}$$

The initial and boundary conditions for the confined aquifer are as follows.

(37) $$h_{1}\left(r,0\right)=0$$

(38) $$h_{1}\left(\infty ,0\right)=0$$

(39) $$\underset{r\to 0}{\lim}\frac{\partial h_{1}\left(r,t\right)}{\partial r}=-\frac{Q}{2\pi T^{\prime }}$$

The initial and boundary conditions for the aquitard are as follows.

(40) $$h_{2}\left(r,z,0\right)=0$$

(41) $$h_{2}\left(r,0,t\right)=0$$

(42) $$h_{2}\left(r,m_{2},t\right)=h_{1}\left(r,t\right)$$

where *r* is the radial distance from the observation well to the center of the pumping well [L]; *t* is time [*t*]; *z* is the vertical distance from the top of the aquitard [L]; *h*_1_ (*r*, *t*), *T*’, and *S*’ are the water level drawdown [L], hydraulic conductivity [L^2^/T], and water storage rate [L^−1^] of the confined aquifer, respectively; *h*_2_ (*r*, *t*), *m*_2_, *T*, *k,* and *S* are the water level drawdown [L], thickness [L], hydraulic conductivity [L^2^/T], permeability [LT^−1^], and water storage rate [L^−1^] of the aquitard, respectively; *Q* is the pumping rate [L^3^T^−1^].

The Laplace transform is applied to Equation (35), and considering the initial condition *h*_1_ (*r*, 0) = 0, one has

(43) $$L\left(\frac{\partial^{2}h_{1}\left(r,t\right)}{\partial r^{2}}\right)+\frac{1}{r}L\left(\frac{\partial h_{1}\left(r,t\right)}{\partial r}\right)-\frac{k\left(t\right)}{T^{\prime }}L\left(h_{0}-h_{1}\left(r,t\right)\right)=\frac{S^{\prime }}{T^{\prime }}sL\left(h_{1}\left(r,t\right)\right)$$

The properties of the Laplace transform are used to obtain

(44) $$\frac{d^{2}{\bar{h}}_{1}\left(r,s\right)}{{dr}^{2}}+\frac{1}{r}\frac{d^{2}{\bar{h}}_{1}\left(r,s\right)}{dr}-\frac{k\left(s\right)}{T^{\prime }}\left(h_{0}\delta \left(s\right)-{\bar{h}}_{1}\left(r,s\right)\right)=\frac{S^{\prime }}{T^{\prime }}s{\bar{h}}_{1}\left(r,s\right)$$

The boundary conditions can be described as follows:

(45) $${\bar{h}}_{1}\left(r,0\right)=0$$

(46) $${\bar{h}}_{1}\left(\infty ,0\right)=0$$

(47) $$\underset{r\to 0}{\lim}\frac{d{\bar{h}}_{1}\left(r,s\right)}{dr}=-\frac{Q}{2\pi T^{\prime }}$$

The Laplace transform is applied to Equation (36), and considering the initial condition $h_{2}\left(r,z,0\right)=0$, one has

(48) $$T\frac{\partial^{2}{\bar{h}}_{2}\left(r,s\right)}{\partial z^{2}}=Ss{\bar{h}}_{2}\left(r,s\right)$$

The boundary conditions are

(49) $${\bar{h}}_{2}\left(r,0,s\right)=0$$

(50) $${\bar{h}}_{2}\left(r,m_{2},s\right)={\bar{h}}_{1}\left(r,s\right)$$

Solving this equation gives

(51) $$\frac{\partial^{2}{\bar{h}}_{2}\left(r,s\right)}{\partial z^{2}}=\frac{Ss}{T}{\bar{h}}_{2}\left(r,s\right)$$

In order to obtain a time-domain solution for *h*_2_(*r*,*t*), the inverse Laplace transform for $\bar{h_{2}}\left(r,z,s\right)$ is performed:

(52) $${\bar{h}}_{2}\left(r,z,s\right)=\frac{Q}{2\pi T^{\prime }}\frac{1}{s}e^{-r\sqrt{\frac{S^{\prime }s}{T^{\prime }}}}\left(\sin h\left(\sqrt{\frac{Ss}{T}}z\right)/\sin h\left(\sqrt{\frac{Ss}{T}}m_{2}\right)\right)$$

Since the inverse Laplace transform involves complex integration and inverse transform tables,

(53) $$\frac{\partial h_{2}\left(r,z,t\right)}{\partial r}=-\frac{Q}{2\pi T^{\prime }}\sqrt{\frac{S^{\prime }}{T^{\prime }}}\left[\frac{e^{-r}\sqrt{S^{\prime }t/T^{\prime }}}{\sqrt{\pi t}}\left(\sin h\left(\sqrt{\frac{S}{T}\cdot t}z\right)/\sin h\left(\sqrt{\frac{S}{T}\cdot t}m_{2}\right)\right)\right]$$

Therefore, the aquitard drawdown is

(54) $$\frac{\partial h_{2}\left(r,t\right)}{\partial r}=-\frac{Q}{2\pi T^{\prime }}\sqrt{\frac{S^{\prime }}{T^{\prime }}}\left[\frac{e^{-r}\sqrt{S^{\prime }t/T^{\prime }}}{\sqrt{\pi t}}\left(\sin h\left(\sqrt{\frac{S}{T}\cdot t}\cdot z\right)/\sin h\left(\sqrt{\frac{S}{T}\cdot t}{\cdot m}_{2}\right)\right)\right]$$

From $v=-k\frac{\partial h}{\partial r}$, it is given that

(55) $$v=\frac{kQ}{2\pi T^{\prime }}\sqrt{\frac{S^{\prime }}{T^{\prime }}}\left[\frac{e^{-r}\sqrt{S^{\prime }t/T^{\prime }}}{\sqrt{\pi t}}\left(\sin h\left(\sqrt{\frac{S}{T}\cdot t}\cdot z\right)/\sin h\left(\sqrt{\frac{S}{T}\cdot t}{\cdot m}_{2}\right)\right)\right]$$

#### 2.1.4. Contaminant Migration Equation with Variable Parameters

Substituting Equations (34) and (55) into (25) obtained the control equation for the transport of NAPL as follows:

(56) $$C\left(r,z,t\right)=L^{-1}\left[A_{1}e^{\sqrt{\frac{\lambda^{2}}{D_{z}s}}z}+A_{2}e^{\sqrt{\frac{\lambda^{2}}{D_{z}s}}z}+B_{1}J_{0}\left(\sqrt{\frac{R+\lambda^{2}}{D_{r}}}r\right)+B_{2}Y_{0}\left(\sqrt{\frac{R+\lambda^{2}}{D_{r}}}r\right)\right]$$

where $J_{0}\left(x\right)=1-\frac{x^{2}}{4}$, $Y_{0}\left(x\right)=-\frac{2}{\pi }ln\left(x\right)+\Upsilon$; Υ is a Euler constant.

In summary, by applying the Laplace transform and the separation variables method to the NAPL concentration control Equation (1), the one-dimensional consolidation deformation Equation (26), and the groundwater flow Equation (36), the relationship between NAPL concentration in the aquitard and various parameters was obtained. It is speculated that the migration laws of NAPLs in the aquitard is affected by a variety of parameters, including the pore water pressure, porosity, permeability, consolidation deformation, and groundwater flow rate of the aquitard, which are determined through laboratory experiments and field tests. Continuous pumping during the experiments caused the relevant parameters of the aquitard to change over time. The migration law of solutes in the aquitard is mainly affected by the porosity and the groundwater flow rate. During the experiment, the porosity can be determined by monitoring the pore water pressure and consolidation deformation, and the groundwater flow rate can be determined by the pumping rate. This is because most parameters cannot be obtained directly through the experimental process, and the detection of these parameters plays a vital role in studying the migration laws of concentration in the aquitard. In order to verify the validity and accuracy of the proposed analytical solution, this study selected the pore water pressure, consolidation deformation of the aquitard, and the groundwater flow rate as the research objects to explore their influence on the migration laws of LNAPLs in the aquitard.

### 2.2. Experimental Materials and Design

#### 2.2.1. Design of Experimental Model

**Figure 2:**
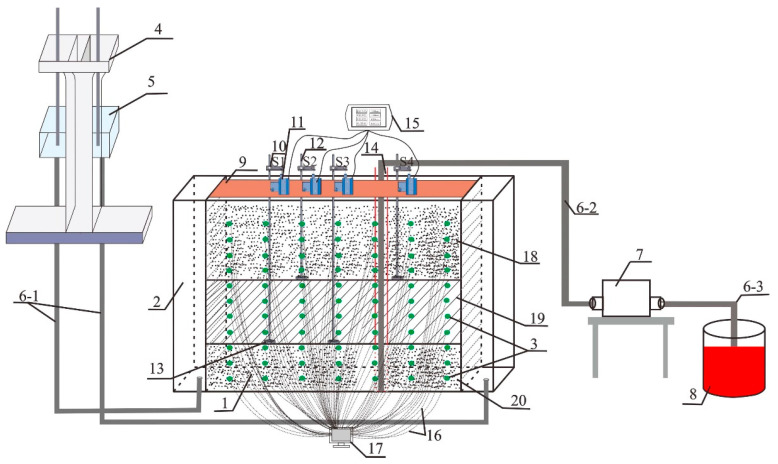
Schematic diagram of the experimental apparatus (1 Experimental box, 2 Inlet and outlet tanks, 3 Monitoring holes (green dots), 4 Overflow device, 5 Overflow device tank, 6 Pressure transfer hoses, 7 Peristaltic pump, 8 Water storage bucket, 9 Settlement fixing platform, 10 Rigid bracket, 11 Pull rope displacement sensor, 12 Rigid bar, 13 Rigid permeable base, 14 Pumping well (red lines), 15 Serial screen, 16 Wire, 17 Computer console, 18 Unconfined aquifer, 19 Aquitard, 20 Confined aquifer).

The experimental apparatus was a rectangular tank constructed from plexiglass with dimensions of approximately 1.0 m × 0.1 m × 0.6 m. The tank comprised three parts. The central section of the box, which was 0.8 m long, formed the main body of the experimental setup. The experimental material was filled in the central part of the box. The remaining two parts were positioned on both sides of the experimental apparatus and served as water tanks, each having a length of 0.1 m (Figure 2). The water tank was connected to the overflow box from the bottom to regulate the experimental water head size. The overflow box, linked to the peristaltic pump, served to stabilize the experimental water head during the pumping process. In the central main section of the experimental apparatus, 77 monitoring holes were correspondingly opened on both sides. These holes primarily functioned as observation points, contaminant injection points, and sampling locations. The horizontal distance between holes was 0.1 m, and the vertical distance was 0.05 m (Figure 2).

The aquifer studied in the experiment was a three-layer aquifer system including unconfined aquifer, aquitard, and confined aquifer. These types of aquifer structures are often found in the Yangtze River Delta region of China, the Edo River region of Japan, and parts of the capital city of Mexico, among others [39]. The lower and upper layers of the experimental setup were placed with standard sand with an initial hydraulic conductivity ranging from 2.31 × 10^−3^ to 5.79 × 10^−5^ m/s. The middle layer was filled with a mixture of quartz sand and kaolinite in a mass ratio of 8:2, creating a ‘sandwich’ structure. This middle layer had an initial hydraulic conductivity ranging from 2.86 × 10^−7^ to 5 × 10^−8^ m/s. To ensure the uniformity of the filling medium, at every 0.02 m height of filling, the outer wall was gently compacted and tapped using cardboard to maintain a tight sand layer. The middle layer, serving as the aquitard, had a thickness of 0.2 m. The confined aquifer in the lower layer had a thickness of 0.15 m, and the unconfined aquifer in the upper layer had thicknesses ranging from 0.15 to 0.25 m. The aquifer structure was set up using detailed geological information obtained from the geological survey. The factors affecting the migration of contaminants were simulated and analyzed in the experimental setup. The results of the experiments were summarized in order to avoid possible accidents during the implementation of the project. The migration mechanism of LNAPL in the aquitard was studied through indoor experiments and theoretical derivation to determine its migration characteristics and provide reference for field tests and site remediation studies.

#### 2.2.2. Observation of the Pore Water and Consolidation Deformation

The experimental device was equipped with 77 monitoring holes, the pressure sensors (XGZP6869A, Wuhu CFSensor Co., Ltd., Wuhu, Jiangsu Province, China), and an automatic high-frequency data collection system (ADS1256-24ADC, Shenzhen Xuanwei Electronic Technology Co., Ltd., Shenzhen, Guangdong Province, China) monitored absolute pressure at different positions in the aquitard at numbered ports, which were connected to the computer for real-time monitoring.

The rigid device consisted of a rigid permeable base, a rigid bar, and a rigid bracket, forming a whole. The rigid bracket was also connected to a drawstring displacement sensor (BRT38-RS485, Shenzhen Briter Technology Co. Ltd., Shenzhen, Guangdong Province, China), connecting the sensors to a serial screen with readings reflecting the amount of consolidation deformation in the aquifer during the experiment. All the above equipment constituted the experimental setup for consolidation deformation monitoring in the experiment. As shown in Figure 2, S1, S2, S3 and S4 were the numbers of the four drawstring displacement sensors, respectively (Figure 2). S1 and S3 were used to monitor the consolidated deformation of the confined aquifer, and S2 and S4 were used to monitor the sum of the consolidated deformations of the aquifer and the confined aquifer. Therefore, the monitored consolidation deformation of the aquifer was the deformation monitored by S2 or S4 minus the deformation monitored by S1 or S3. The rigid device base was placed at the bottom of the measured aquifer and at the top of the measured aquifer, and then the detection value at the top was subtracted from the detection value at the bottom to obtain the consolidation deformation of the measured aquifer. When considering the layered monitoring of the aquitard, the rigid base was placed at the layered interface of the aquitard to obtain the consolidation deformation of the aquitard.

#### 2.2.3. Contaminant Injection and Sampling Analysis

No. 0 diesel was chosen as the representative contamination for LNAPL in this experiment. LNAPL was injected into the hole O as the contamination source. The initial concentration of LNAPL was 0.56 mg/L. Following the initiation of the pumping experiment, samples of the contaminant were collected from the observation hole every 10 min, numbered, and sealed for storage. These samples were analyzed within 72 h of collection. The concentration of LNAPL in the exudate was measured using a Model 754 (automatic) UV spectrophotometer manufactured by Shanghai Jinghua Instrument Factory, Shanghai, China, according to laboratory protocols.

**Figure 3:**
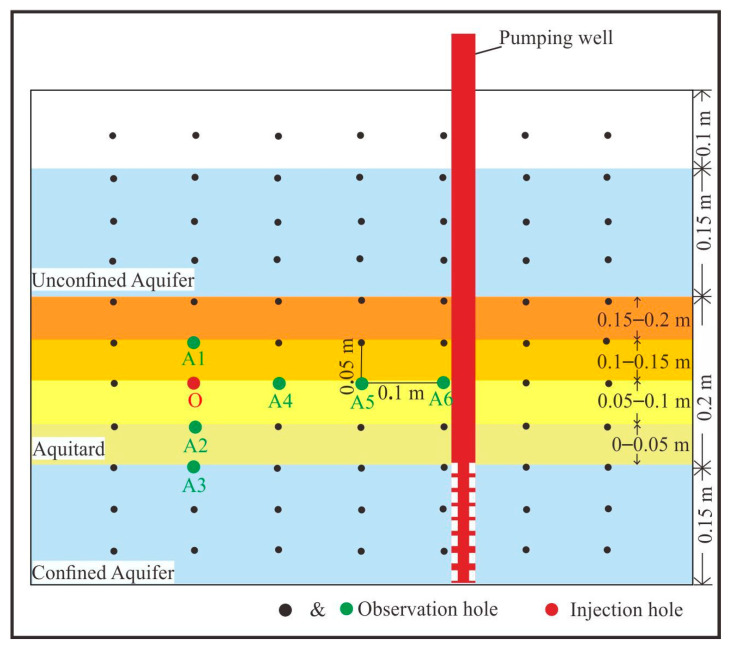
Locations of observation holes and injection hole.

As shown in Figure 3 and Table 1, the hole O was the injection hole, located 10 cm from the bottom of the aquitard and 30 cm from the pumping well; the holes A1, A2, A3, A4, A5, and A6 were observation holes.

#### 2.2.4. Pumping Simulation

Peristaltic pumps (BT300-2 J, Longer Precision Pump Co., Ltd., Baoding, Hebei Province, China) were employed as pumping devices to facilitate precise control of both the volume and pumping rate. The pumping well, constructed from Poly Vinyl Chloride (PVC) hard pipe with a diameter of 0.04 m, featured evenly distributed holes along a length of 0.15 m from one end of the pipe. A screen was positioned at the opening, and the PVC pipe was embedded in the experimental apparatus during the pumping test, with the open end of the hole corresponding to the pressurized aquifer. This research employed a constant flow rate, with the pumping rate controlled by adjusting the revolutions of a peristaltic pump. The pumping rate can be set to 3, 6, 9, 12 mL/s.

## 3. Results and Analysis

To validate the proposed analytical method, laboratory experiments were used. The correlation between the pore water pressure, consolidation deformation, and pumping rate were explored by monitoring or designing for the laws of LNAPL migration in the aquitard.

### 3.1. Effect of the Pore Water Pressure on LNAPL Migration

The water level changes due to pumping, and the drawdown of the water level is continuous between the confined aquifer and the aquitard. It leads to changes in the stress state in the aquitard. The increase can be caused by the stress between the solid skeleton particles in the aquitard. Based on the curve of the pore water pressure with time in the aquitard, it can be seen that after the start of pumping, there is a transient change in the pore water pressure of the aquitard due to the change in water level caused by pumping. The pore water pressure rises to a maximum in a short period of time. In the vicinity of the pumping wells, the effect on pore water pressure is significant. The closer the observation holes are to the pumping wells, the more promptly they respond to changes in pore water pressure, reaching the peak pressure earlier. As the pumping duration increases, the pore water pressure gradually dissipates, leading to consolidation deformation in the soil body.

**Figure 4:**
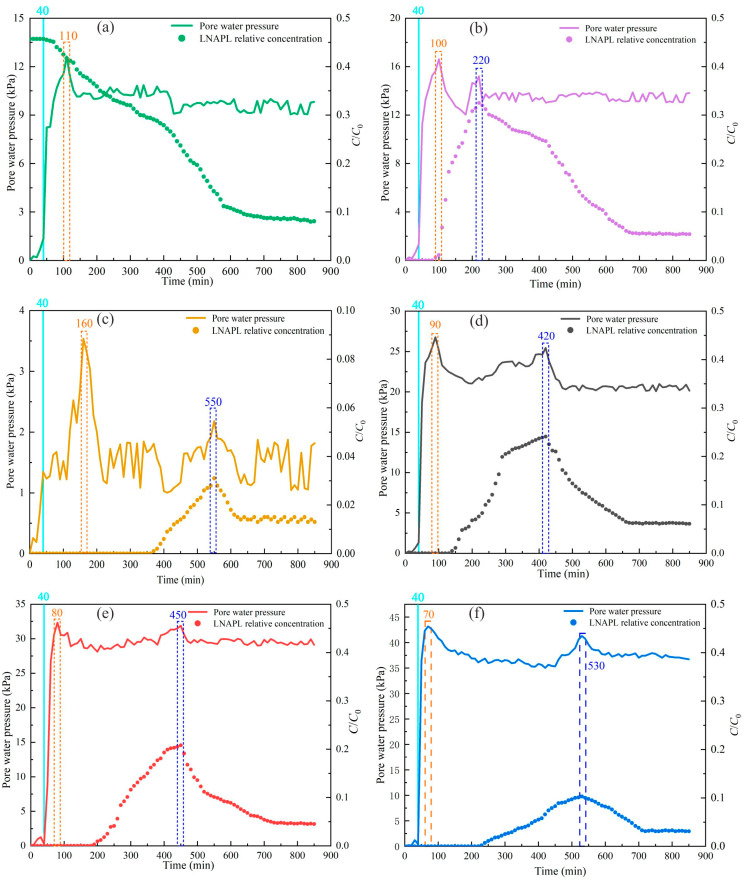
Dynamic variations in the pore water pressure and LNAPL relative concentration at different locations ((**a**) the monitoring hole O, (**b**) the hole A1, (**c**) the hole A2, (**d**) the hole A4, (**e**) the hole A5, (**f**) the hole A6).

As shown in Figure 4, the observation holes closest to the pumping well were the first to respond to changes in pore water pressure. Among them, A5, being the nearest to the pumping well, exhibited the promptest response and the greatest magnitude of change in pore water pressure. Observation holes A1 and A2 were situated on the upper and lower sides of injection hole O, respectively, while observation holes A3, A4, and A5 were positioned on the right side of the injection hole. As the pumping test progressed, the LNAPL contaminants spread in all directions from the injection point, which served as the center of the leak. However, the rate and extent of LNAPL migration varied in different directions.

In Figure 4a, the first peak is the response to pore water pressure at different locations after the start of pumping. The concentration of LNAPLs in injection hole O gradually decreases with increased pumping time. The pore water pressure in injection hole O also shows a steady decline during the pumping test, with no secondary peak observed in the pore water pressure. As shown in Figure 4b–f, observation hole A1 detected the signal of LNAPL migration at 220 min, hole A3 reached its second peak at 330 min, and hole A2 did not reach its peak until 550 min. This indicated that due to the lower specific gravity of LNAPLs compared with water, downward migration in the aquitard was hindered, with predominant migration occurring toward the pumping well and upward. Observation holes A3, A4, and A5 were positioned at distances of 10 cm, 20 cm, and 30 cm, respectively, from the right side of the injection hole. The time to detect the LNAPL migration signal increased gradually with greater distance from the injection hole. Monitoring changes in pore water pressure in the aquitard allowed for tracking the process and trends of LNAPL migration.

The nonlinear distribution here can be regarded as the result of a dynamic balance between the decrease in pore water pressure caused by groundwater extraction and the increase in pore water pressure caused by creep extrusion. The more obvious the rheological characteristics are, the more obvious the nonlinear distribution of pore water pressure is. And the pore water pressure indirectly affects the change in NAPL concentration. This is consistent with the analysis of the change in influencing factors related to NAPL concentration described by the variable parameter contaminant migration Equation (56).

### 3.2. Effect of the Consolidation Deformation on LNAPL Migration

**Figure 5:**
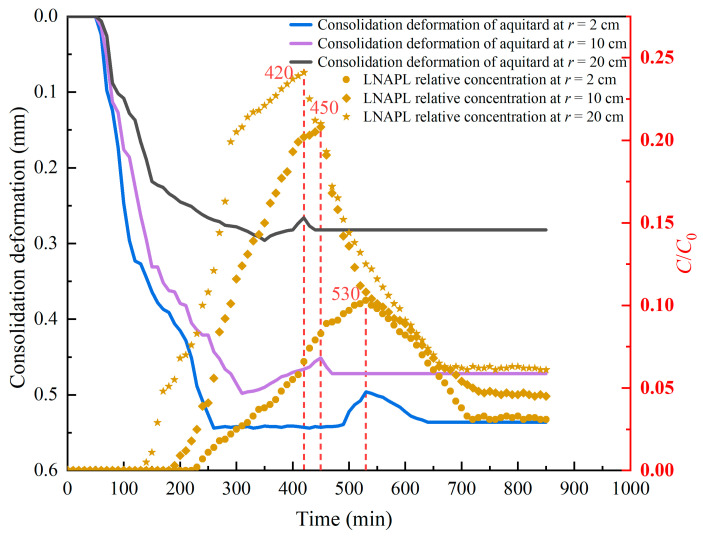
Horizontally oriented consolidation deformation and LNAPL relative concentration in the aquitard during pumping.

According to the effective stress principle, when the aquitard releases water, the pore water pressure decreases, the effective stress in the soil increases, and the soil undergoes compression deformation. In order to study the effect of consolidation deformation on LNAPL migration, the effect of consolidation deformation in the aquitard on LNAPL migration was analyzed from the horizontal and vertical directions of the aquitard. The initial water head was 50 cm, the pumping rate was 12 mL/s, and the pumping was continued for 850 min. Other conditions followed the above. The consolidation deformation curve and the change in LNAPL relative concentration over time at a distance of 10 cm from the bottom of the aquitard and *r* = 2, 10, and 20 cm from the pumping well were plotted (*r* is distance from the pumping well), as shown in Figure 5. The change in consolidation deformation in the horizontal direction of the aquitard during pumping was analyzed, and the migration trajectory and migration law of LNAPL were inferred based on the change in LNAPL concentration in the observation hole during this process.

Figure 5 shows that the slope of the curve was large at the initial stage of consolidation deformation of the aquitard. As the aquitard settled, the curve gradually slowed down and the slope gradually decreased. The closer to the axis of the pump well, the greater the effect of pumping on the change in consolidation deformation. The maximum settlement occurred near *r* = 2 cm, and the maximum consolidation deformation was 0.544 mm. As the distance from the pumping well increased, the consolidation deformation of the aquitard gradually decreased. The consolidation deformation at *r* = 20 cm was 0.296 mm. As LNAPL contaminants migrated toward the pumping well along with the water flow, the aquitard had a temporary rebound phenomenon when it reached the observation hole, and the consolidation deformation of the aquitard decreased during the rebound process. As shown in Figure 5, when the consolidation deformation of the aquitard rebounded, the relative concentration of LNAPL in the aquitard reached the maximum value. The concentrations migrated from the injection hole (30 cm away from the pumping well) to the pumping well, and the LNAPL relative concentration decreased as the distance from the pumping well decreased. At *r* = 20 cm, the maximum LNAPL relative concentration in the aquitard reached 0.241. However, when migrating to *r* = 2 cm, the LNAPL relative concentration finally decreased to 0.031.

**Figure 6:**
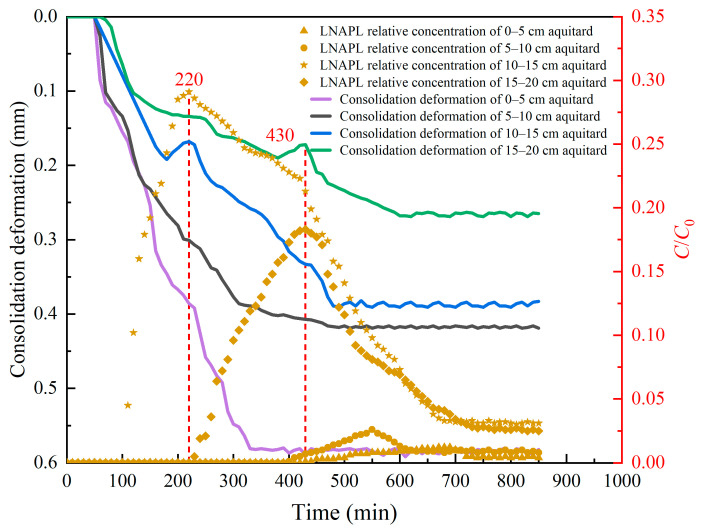
Vertically oriented consolidation deformation and LNAPL relative concentration in the aquitard during pumping.

Figure 6 shows the distribution of consolidation deformation and LNAPL relative concentration in the different aquitard layers. As can be seen from Figure 6, the consolidation deformation of the aquitard gradually decreased from the bottom to the top. The maximum consolidation deformation in the 0–5 cm layer of the aquitard reached 0.590 mm, and the maximum consolidation deformation in the 15–20 cm layer of the aquitard was 0.269 mm. Pumping caused consolidation deformation in the aquifer. This was because the load transfer direction was from bottom to top. The effective stress increase was the largest at the pumping point. The closer to the ground, the smaller the water level drop, and the smaller the effective stress increase. As shown in Figure 3, the LNAPL injection hole was located at the top of the 5–10 cm aquitard. As shown in Figure 6, the LNAPL residual amounts in the 0–5 cm and 5–10 cm layers of the aquitard were small, and the relative concentration of LNAPL in the 5–10 cm layer reached a maximum value of 0.026 at 550 min. The relative concentration of LNAPL in the 10–15 cm layer of the aquitard was the largest, reaching 0.291, and the maximum relative concentration of LNAPL in the 15–20 cm layer was 0.183. This phenomenon was related to the physical properties of LNAPL. The density of LNAPL is less than that of water. The LNAPL injected into the aquitard showed the phenomenon of upward migration.

Comparing Figure 5 and Figure 6, as the pumping test started, LNAPL mainly migrated toward the pumping well and upward. The consolidation deformation at the bottom of the aquitard near the pumping well was the largest, and the consolidation deformation gradually decreased from the well axis to the outside and from the bottom of the aquitard to the top. The LNAPL relative concentration in the aquitard reached the maximum value during the transient rebound stage of the consolidation deformation. The figure shows the rebound time and the change in the relative concentration of LNAPL, which illustrate the great influence of the consolidation deformation induced by pumping on the migration characteristics of LNAPL. In addition, it can be seen from the figure that whether it is the horizontal direction or the vertical direction of the aquitard, the consolidation deformation and the relative concentration of LNAPL always show a good consistency. Therefore, the relative migration trajectory of LNAPL can be preliminarily determined according to the position where the consolidation deformation rebounds during the pumping process. It can be seen from the control Equations (33) and (56) that the amount of settlement deformation indirectly affects the NAPL concentration by affecting the size of the porosity. The larger the amount of settlement deformation, the smaller the porosity is relatively, and the correspondingly smaller the NAPL concentration is. This is similar to the analysis obtained from Figure 5 and Figure 6.

### 3.3. Effect of the Pumping Rate on LNAPL Migration

During the pumping test, the water level in the pumping well dropped rapidly, resulting in a head difference inside and outside the pumping well. The groundwater then flowed into the pumping well and was pumped away by the pump. At the specified constant head boundary, constant flow pumping was used to explore the effect of different pumping rates on the relative concentration migration of LNAPL in the aquitard. The pumping well flow rate could be controlled by the number of revolutions of the peristaltic pump to simulate the pumping of the confined aquifer. It is also evident through Equation (55) that there is a positive proportional relationship between groundwater flow rate and pumping flow rate. During the experiment, the initial head of the experiment was 50 cm, and the pumping time was set to 850 min. The pumping rates were set to 3 mL/s, 6 mL/s, 9 mL/s, and 12 mL/s. The pumping test caused the groundwater level to change, and the change in the water level of the aquitard caused a hydraulic gradient, which triggered the seepage consolidation in the aquitard system. The faster the pumping rate, the greater the water level difference and the corresponding LNAPL migration rate. From Table 2, it can be seen that the average water level differences generated when the pumping rates were 3 mL/s, 6 mL/s, 9 mL/s, and 12 mL/s were 3.56, 13.68, 19.12, and 39.29 cm, respectively.

**Figure 7:**
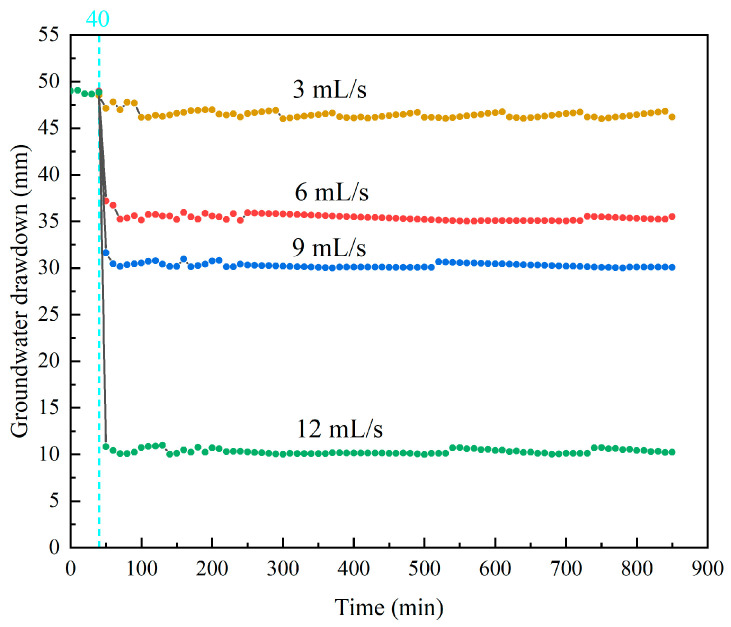
Dynamic variations in groundwater drawdown at different pumping rates.

As shown in Figure 7, the water level changes in the four pumping rates were quite different at the beginning of pumping, and the greater the pumping rate, the greater the water level change. After reaching a certain water level drop, the water level difference caused by the four pumping rates reached a balanced state.

**Figure 8:**
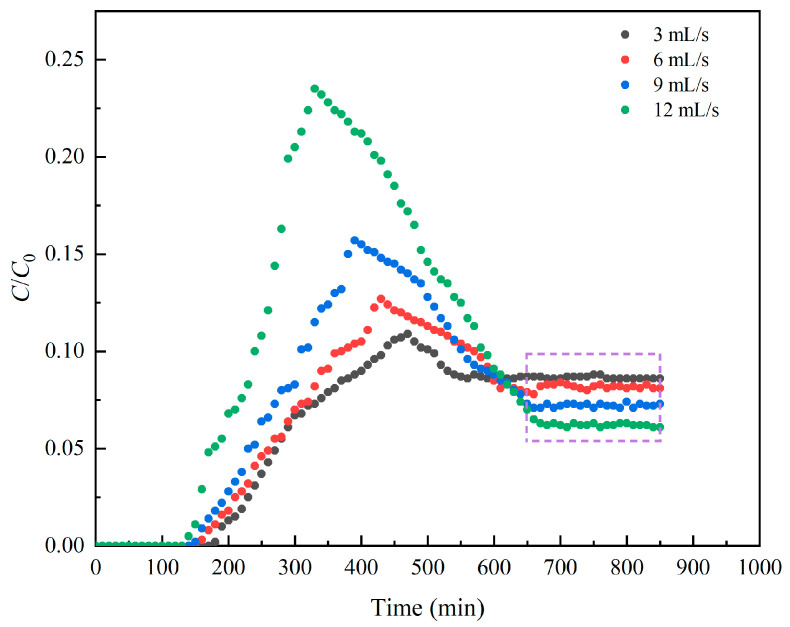
Dynamic variations in LNAPL relative concentrations at different pumping rates in hole A4.

Figure 8 shows the relative concentration change curve of LNAPL in hole A4 at different pumping rates. The development trend of LNAPL migration under different pumping rates was similar. At the same time, the faster the pumping rate, the greater the relative concentration of LNAPL. However, the pumping rate had different effects on the start time and migration rate of LNAPL migration. When the pumping rate was 12 mL/s, the migration rate of LNAPL in the aquitard was the fastest. This was because the increase in pumping rate accelerated the migration of groundwater toward the pumping well, causing more LNAPL to be discharged with pumping. When the pumping rate was 12 mL/s, the migration trajectory of LNAPL was observed earlier in the monitoring hole A4. From Equation (56), it can be concluded that groundwater flow rate has a positive correlation on NAPL concentration. The results of the experimental analyses coincide with the derived equations.

As the pumping time increased, the relative concentration of LNAPL residue in the aquitard of the experimental group with a larger pumping rate became lower, but the time to reach the lowest relative concentration became longer. This was because as the pumping test time increased, the soil layer of the aquitard of the experimental group with a faster pumping rate reached a dense state earlier, and the LNAPL in the aquitard was more difficult to discharge. As shown in Figure 8, when the pumping rate was 12 mL/s, the aquitard approached the final residual concentration at 660 min. When the pumping rate was 3 mL/s, the aquitard had reached the final residual concentration at 550 min.

## 4. Discussion

The expression for the concentration of LNAPL in the aquitard during pumping was proposed. It can be seen that it was affected by the multiple parameters of the aquitard. Based on previous research results, pumping water would significantly cause the parameter variations in the aquitard [29]. The effects of the pore water pressure, consolidation deformation, and pumping rate were considered in this study. However, other details, such as the porosity, hydraulic conductivity, specific storage, and compression coefficient of the aquitard were not included. This was based on the consideration of data availability during the experiment.

First, pumping caused the changes in the pore water pressure in the aquitard [40]. The first peak of the pore water pressure appeared in the early stage of pumping. As the LNAPL migrated, when the LNAPL reached the observation hole, the pore water pressure at the observation hole reached the second peak. This is because the migration of LNAPL to the observation hole affected the overall stress state, and the original simple pore water pressure was converted into a stress combined with pore water and LNAPL. Secondly, according to Galloway and Burbey [41], when the groundwater level drops, the decrease in pore water pressure increases the effective stress of soil particles, causing deformation of the aquifer. The effects of consolidation deformation and pore water pressure on LNAPL migration had certain similarities. These factors first affected the vicinity of the pumping well, and then, the impact was transferred to a location farther away from the pumping well. The change in consolidation deformation over time was presented as a steady increase first, and then a gradually stable state. Third, it was generally believed that a constant pumping rate is the ideal state. However, as time went by, the pressure drops in the pumping well increased, making it more difficult to pump water from the pumping well [42,43]. Increasing the pumping rate could increase the groundwater flow rate in the aquitard, and thus, accelerate the dissipation of LNAPL. This was similar to the three-aquifer study of Feng et al. [44], which used a constant pumping rate.

The influence mechanisms of these factors were identified that could be used to predict the migration laws of LNAPL in the aquitard. In order to eliminate LNAPL in the aquitard, the location for the pumping well should be selected to be as close to the contaminated area as possible. For the pumping rate, which is artificially controllable, the pumping rate could be accelerated for a certain period of time in order to accelerate the elimination of LNAPL in the aquitard. This study explored the change mechanism of the parameters of the aquitard during the pumping process and conducted a detailed analysis and discussion of the impact of LNAPL migration. It is necessary to further explore the impact of the precipitation schemes, the burial depth of the aquitard, and the hydraulic conductivity of the aquitard on the migration laws of LNAPLs.

## 5. Conclusions

In this study, an analytical model for contaminant migration was developed by considering the one-dimensional consolidation equation of the aquitard and the groundwater flow equation. Based on the priority of confirming the changes in various parameters in the aquitard during the pumping process, a method for determining the concentration of NAPL contaminants in the aquitard was proposed. Through the derived equations for the migration of NAPLs in the aquitard, the main factors affecting the migration of contaminants were deduced to provide an effective reference for indoor experiments. This method was then verified by the laboratory experiments.

The experimental device and platform integrated multiple monitoring systems for monitoring contaminant migration in the aquitard, which could obtain real-time data such as the pore water pressure, consolidation deformation, and water level changes in the aquitard. Through a series of laboratory experiments, a better understanding of the changes in pore water pressure and consolidation deformation in the aquitard during pumping could be obtained, which could help us understand in detail the migration mechanism of LNAPLs in the aquitard during pumping. In addition, the effect of groundwater flow rate on LNAPL migration in the aquitard was studied based on the setting of the pumping rate. The conclusions of this study were as follows:

(1) The pore water pressure in the aquitard increased rapidly at the beginning of the pumping test and tended to stabilize as the pumping proceeded. When the pore water pressure reached the second peak, it indicated that the LNAPL in the aquitard had migrated to the observation location. Based on the second peak time of the pore water pressure at each observation point, the overall migration trend of the LNAPL in the aquitard could be easily identified.

(2) The stratified settlement of the aquitard caused by pumping showed a pattern of being small at the top and large at the bottom, which is not the traditional pattern of large at the top and small at the bottom caused by soil surface loading. The closer the distance was to the pumping well, the greater the consolidation deformation, and the closer the stratified consolidation deformation was to the bottom of the aquitard, the greater the consolidation deformation. The influence of consolidation deformation on LNAPL migration was reflected in the rebound of consolidation deformation. The consolidation deformation of the aquitard was almost the same as the development law of the pore water pressure. The greater the pressure reduction, the greater the compaction of the aquitard. The migration trajectory of LNAPL in the aquitard could be roughly determined by the time of consolidation deformation rebound at each position in the aquitard.

(3) In the experiments, the groundwater flow rate in the aquitard was mainly affected by the pumping rate. The effect of four pumping rates on the migration of LNAPL in the aquitard was well discussed. It showed that the greater the pumping rate, the faster the migration of LNAPL in the aquitard and the lower the relative concentration of LNAPL remaining in the aquitard.

The one-dimensional theoretical derivation obtained in this paper was verified through a series of indoor LNAPL migration experiments. The theoretical study of contaminant migration in the aquitard was enriched to provide a basis for the discrimination of contaminant flow direction at the site. The results of the experiments can provide guidance for the arrangement of groundwater monitoring points at a site as well as provide a scientific basis for the remediation of contaminated sites to reduce remediation costs. The theoretical formulation of two-dimensional and three-dimensional equations will be carried out on the basis of the one-dimensional equations in future research.

## Figures and Tables

**Table 1:** Characteristics of experimental holes.

Label of Hole Number	Distance from the Bottom of the Aquitard (cm)	Distance from Pumping Well (cm)	Type
O	10	30	Injection hole
A1	15	30	Observation hole
A2	5	30	Observation hole
A3	0	30	Observation hole
A4	10	20	Observation hole
A5	10	10	Observation hole
A6	10	2	Observation hole

**Table 2:** Settings of experimental parameters.

Scenario	Pumping Rate (mL/s)	Initial Water Level (cm)	Groundwater Flow Rate (m/s)	Average Water Level Difference (cm)	LNAPL Start Migration Time	Time to Maximum LNAPL Relative Concentration
Sce. 1	3	50	2.39	2.19	180	330
Sce. 2	6	50	4.77	12.81	160	390
Sce. 3	9	50	7.16	17.84	150	430
Sce. 4	12	50	9.55	38.67	140	470

## Data Availability

The original contributions presented in the study are included in the article; further inquiries can be directed to the corresponding author.

## Abbreviations

The following abbreviations are used in this manuscript:

LNAPL	Light Non-aqueous Phase Liquid
NAPL	Non-aqueous Phase Liquid
PVC	Poly Vinyl Chloride
P&T	pump-and-treat technology
Notations	
*C*	concentration of Non-aqueous Phase Liquid
*C*_0_	initial concentration of Non-aqueous Phase Liquid
*r*	radius distance from the center of the well
*rw*	radius of the well
*z*	vertical distance
*v*	groundwater flow rate
*Dz*	radial dispersion coefficient
*Dr*	vertical dispersion coefficient
*D*_0_	effective diffusion coefficient
*Da*	free water molecular diffusion coefficient of the solute
*ar*	radial dispersivity values
*az*	vertical dispersivity values
*R*	retardation factor of the aquitard
*T*	thickness
*h*	water level drawdown
*kd*	equilibrium distribution coefficient
*ρb*	bulk density of the aquitard material
*θ*	porosity in the aquitard
*Q*	pumping rate
*s*	Laplace transform parameter with respect to t
*t*	time
*Cv*	consolidation coefficient
*u*	pore water pressure in the aquitard
*γw*	weight of water
*e*_0_	initial void ratio of the aquitard
*k*_0_	initial hydraulic conductivity of the aquitard
*k*	hydraulic conductivity
*a*	compression coefficient of the aquitard
*sc*	consolidation deformation
*S*	water storage rate
